# Role of Diet in Chronic Obstructive Pulmonary Disease Prevention and Treatment

**DOI:** 10.3390/nu11061357

**Published:** 2019-06-16

**Authors:** Egeria Scoditti, Marika Massaro, Sergio Garbarino, Domenico Maurizio Toraldo

**Affiliations:** 1 National Research Council (CNR), Institute of Clinical Physiology (IFC), 73100 Lecce, Italy; marika.massaro@ifc.cnr.it; 2 Department of Neuroscience, Rehabilitation, Ophthalmology, Genetics and Maternal/Child Sciences, University of Genoa, 16132 Genoa, Italy; sgarbarino.neuro@gmail.com; 3 Rehabilitation Department, Cardio-Respiratory Care Unit, “V Fazzi” Hospital, ASL Lecce, 73100 Lecce, Italy; d.torald@tin.it

**Keywords:** antioxidant, chronic obstructive pulmonary disease, dietary pattern, inflammation, lung function, Mediterranean diet, nutrition, oxidative stress, polyphenol, polyunsaturated fatty acid

## Abstract

Chronic obstructive pulmonary disease is one of the leading causes of morbidity and mortality worldwide and a growing healthcare problem. Identification of modifiable risk factors for prevention and treatment of COPD is urgent, and the scientific community has begun to pay close attention to diet as an integral part of COPD management, from prevention to treatment. This review summarizes the evidence from observational and clinical studies regarding the impact of nutrients and dietary patterns on lung function and COPD development, progression, and outcomes, with highlights on potential mechanisms of action. Several dietary options can be considered in terms of COPD prevention and/or progression. Although definitive data are lacking, the available scientific evidence indicates that some foods and nutrients, especially those nutraceuticals endowed with antioxidant and anti-inflammatory properties and when consumed in combinations in the form of balanced dietary patterns, are associated with better pulmonary function, less lung function decline, and reduced risk of COPD. Knowledge of dietary influences on COPD may provide health professionals with an evidence-based lifestyle approach to better counsel patients toward improved pulmonary health.

## 1. Introduction

Chronic obstructive pulmonary disease (COPD) is a major cause of morbidity and mortality and healthcare burden worldwide, affecting around 10% of the adult populations aged 40 years and older [1]. According to WHO estimates mainly from high-income countries, 65 million people have moderate to severe COPD, but a great proportion of COPD worldwide may be underdiagnosed, mostly in low- and middle-income countries. COPD burden is projected to dramatically increase due to chronic exposure to risk factors and the changing age structure of the world population and is expected to be the third leading cause of death worldwide by 2030 (WHO 2019. Burden of COPD https://www.who.int/respiratory/copd/burden/en). Therefore, prevention and management of COPD is currently considered a major health problem, with important social and economic issues.

COPD encompasses a group of disorders, including small airway obstruction, emphysema, and chronic bronchitis, and is characterized by chronic inflammation of the airways and lung parenchyma with progressive and irreversible airflow limitation [2]. Symptoms of COPD include dyspnea (distress with breathing), cough, and sputum production. The natural history of COPD is punctuated by recurrent episodes of acute exacerbations, which often require hospitalization and negatively affect patients’ quality of life, accelerate the rate of decline in lung function, and are associated with mortality.

Diagnosis, assessment, and management of COPD are mostly guided by the degree of airflow limitation as assessed by the forced expiratory volume in one second (FEV1), forced vital capacity (FVC), and FEV1/FVC ratio, although other physiological measurements such as the inspiratory capacity to total lung capacity (TLC) ratio, arterial blood gases, and exercise capacity provide complementary information on the severity of the disease [3]. To account for the complexity of the disease and aiding in disease severity assessment, multidimensional indices mainly based on clinical and functional parameters have been developed. However, significant heterogeneity in terms of clinical presentation, physiology, imaging, response to therapy, lung function decline, and survival exists in COPD, challenging the oversimplification regarding definition and assessment of COPD and leading to effort in identifying subgroups of patients called phenotypes, resulting from different endotypes (biologic mechanisms) and displaying distinct prognostic and therapeutic value. Accordingly, several COPD phenotypes have been recently described, which exhibit significant differences in age, symptoms, co-morbidities, and predicted mortality [4,5,6,7]. Most studies described COPD heterogeneity using a limited range of variables, and in some cases the clinical relevance of identified phenotypes needs to be determined [8]. Despite these current limitations, the phenotypic characterization of COPD patients with insight into the underlying biological processes and related biomarkers may ultimately allow for a better risk stratification and personalization of therapies [9].

The predominant risk factor for COPD development is former or current tobacco smoking. However, not all smokers develop COPD, suggesting that other environmental factors are also involved, including outdoor and indoor air pollution (e.g., biomass fuel exposure), occupational hazards, infections, and second-hand smoke during pregnancy or early childhood. Furthermore, genetic susceptibility (e.g., deficiency in α1-antitrypsin) and epigenetic influences have also been implicated in the pathogenesis of COPD [10]. Recent new insights suggest that these different factors may impinge on lung function and reciprocally interact starting early in life (i.e., in utero and during early childhood), thus determining many potential trajectories of the natural course of the disease, which ultimately predispose to the development of COPD and its different clinical appearances as well as of other coexisting chronic diseases in later life [10,11].

With regard to COPD management, the most important public health message remains smoking cessation, but the multifactorial nature of COPD requires attention to other modifiable risk factors. Compared with other chronic diseases with similar burdens on quality of life and healthcare costs, such as cancer and cardiovascular disease (CVD), less is known about how lifestyle factors other than—and independent of—smoking influence pulmonary function and the development of COPD. Diet has been recognized as a modifiable risk factor for chronic diseases development and progression [12], and recent evidence has also increasingly pointed to a role in obstructive lung diseases, including COPD [13,14,15]. Importantly, changes in diet over the past few decades, with decreased consumption of fruits, vegetables, wholegrains, and fish, and increased consumption of processed and refined foods, have been invocated to contribute to the increased prevalence of chronic diseases, including COPD, mainly in developing countries.

Dietary factors may modulate the impact of adverse environmental exposures or genetic predisposition on the lung [16] but can also have direct (protective or harmful) effects on the biological processes involved in lung function, disease development, and outcomes [17,18]. The impact exerted by early-life and cumulative dietary choices on later-life health has been increasingly recognized for respiratory diseases, thus offering a greater window of opportunity for disease prevention [19]. Furthermore, the abnormal nutritional status observed in advanced COPD patients, with unintended weight loss, muscle loss, low fat, and fat-free mass associated with the presence of emphysema is a recognized independent determinant of COPD outcomes and provides targets for nutritional interventions [20]. On the other hand, although the phenomenon of the obesity paradox, i.e., the prognostic advantage of increased body mass index (BMI) in COPD (due to the reduction in static volume), has been reported, the role of abdominal visceral adiposity compared with subcutaneous fat in exacerbating the pro-inflammatory state and the CV risk in patients with COPD deserves clinical attention and treatment [21], mostly because a fat redistribution toward more visceral fat and an associated increased systemic inflammatory status have been shown in mild-to-moderate nonobese patients with COPD compared to control subjects [21].

Therefore, improved understanding of dietary impact on prevention and/or outcomes of COPD may increase scientific and clinical awareness about the importance of nutritional approaches as well as provide directions for future research and strategies to promote lung health and prevent disease onset and progression.

**Figure 1:**
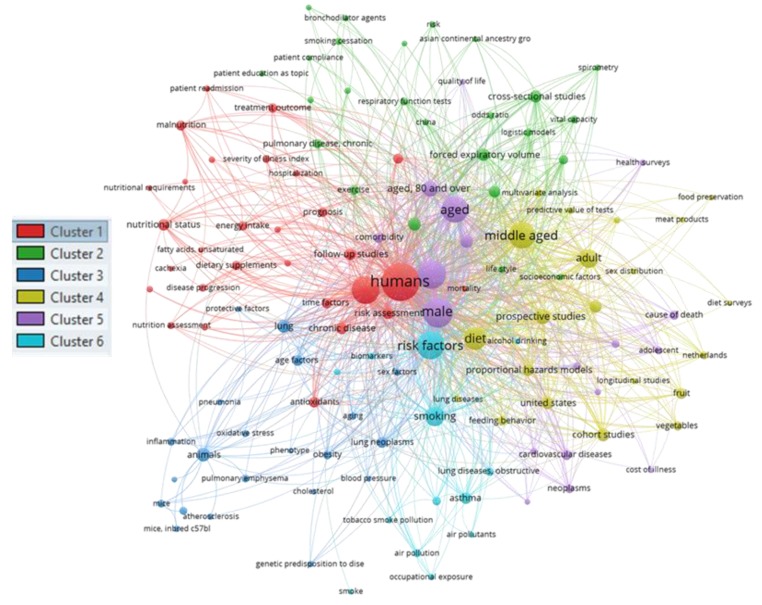
The bubble map visualizes 127 keywords extracted from published papers retrieved in PubMed under the search terms “diet” and “chronic obstructive pulmonary disease risk” between 1989 and 2019. Bubble size indicates the frequency of occurrence of the words, while bubble color represents the cluster of belonging. Words are clustered based on direct citation relations; thus, each cluster corresponds to a set of closely related words. Two bubbles are in closer proximity if the two words had more frequent co-occurrence.

There is an expanding literature on the topic regarding diet–COPD relation. A literature search performed with the PubMed database to identify papers with the following terms “diet” and “chronic obstructive pulmonary disease risk” retrieved 233 manuscripts (from 1989 to 2019). The resulted manuscripts were analyzed using the bio-informatic data analysis tool VOSviewer [22], which extracts and analyzes the words in the titles and abstracts of the publications, relates them to citation counts, and visualizes the results as a bubble or term map, based on the strength of the co-occurrence links within the terms. The terms with greatest total link strength were selected and highlighted as bubbles. The analysis returned 750 words, of which 127 met the threshold levels (minimum number of occurrence of a term = 5). As shown in Figure 1, diet has been the focus of relevant scientific attention, and several of the words retrieved from the analysis were connected to diet, feeding behaviors, and specific foods and nutrients (fruits and vegetables, antioxidants, unsaturated fatty acids, meat products), suggesting some of the main key research categories that have been the attention focus in the topic diet and COPD risk.

The present narrative review aimed at assessing the available evidence from observational and intervention studies to summarize current understanding of the associations between dietary pattern, individual foods, nutrients and lung function, and prevention and improvements of COPD. The benefit of nutritional supplementation (e.g., high protein/high energy) in undernourished COPD subjects is beyond the scope of the present review, and the readers are directed to other papers on the topic (see [20,23] and references therein).

## 2. Pathophysiological Aspects in COPD

Several pathogenic processes are thought to be involved in COPD development and progression, including local and systemic oxidative stress (i.e., oxidants in excess compared with antioxidant capacity) and inflammation (neutrophils, macrophages, eosinophils, cytokines, chemokines, eicosanoids, Toll-like receptors, acute phase proteins), procatabolic status, protease/antiprotease imbalance, alteration of immune responses and cell proliferation, apoptosis, and cellular senescence, and remodeling of the small-airway compartment and loss of elastic recoil by emphysematous destruction of parenchyma [2]. Oxidative stress may directly cause lung damage through modification of DNA, lipids or proteins, as well as initiate cellular responses that can drive the inflammatory response within the lung, leading to lung tissue degradation (emphysema). Molecular switches triggering inflammatory responses in COPD involve the activation of redox-sensitive transcription factors (e.g., nuclear factor (NF)-κB), induction of autophagy, and unfolded protein response [24]. In particular, NF-κB plays a crucial role in the chronic inflammatory responses found in COPD, regulating the expression of genes for pro-inflammatory mediators (e.g., IL-1, IL-6, IL-8, MCP-1, TNF-α) and chemotactic factors (e.g., IL-17A and MIP-1a) involved in triggering lung infiltration by inflammatory cells, thus amplifying oxidative stress and inflammation, as well as causing emphysema, fibrosis of small airways and remodeling of airway walls, ultimately impairing lung function. Indeed, the number of NF-κB-positive epithelial cells and macrophages increased in smokers and COPD patients and correlated with the degree of airflow limitation [25].

Although primarily affecting the lungs, COPD is associated with extra-pulmonary (systemic) manifestations such as weight loss, malnutrition, and skeletal muscle dysfunction, which contribute to the morbidity, reduced quality of life, and, possibly, mortality of this disease. Furthermore, other chronic diseases (also called co-morbidities), including CVD and especially coronary artery disease (CAD), osteoporosis, metabolic syndrome, depression, and lung cancer, among others, are highly prevalent in patients with COPD, can be considered part of the nonpulmonary sequelae of the disease, with the low-grade systemic inflammation playing a decisive role in their pathogenesis, and importantly contribute to worsening health status and vital prognosis of COPD patients. In particular, CV-related co-morbidities are the leading cause of morbidity and mortality in patients with COPD, sharing various risk factors and pathophysiological aspects (inflammation-associated oxidative stress) [26]. Reduced lung function is a marker for all-cause, respiratory- and CV-related mortality [27], thus representing a clinically relevant therapeutic target for preventing the development of COPD and its life-threatening complications.

## 3. Literature Search Strategy

An electronic literature search of MEDLINE/Pubmed, ISI Web of Knowledge, Scopus, EMBASE, and Google Scholar was conducted by two separate investigators to retrieve relevant studies written in English and published between January 1990 and January 2019, using the following keywords: diet, dietary factor, food, nutrition, nutrient, antioxidant, fatty acid, dietary pattern, food pattern, eating habit, lung function, FEV1, chronic obstructive pulmonary disease, COPD. In addition, the references of selected original studies and reviews were scrutinized for further relevant evidence.

## 4. Epidemiological Studies on Diet and Pulmonary Function: Some Methodological Issues

The methodological approaches used and the specific challenges of nutrition research should be taken into consideration when evaluating single study findings and, most importantly, their potential contribution to evidence-based recommendations. Apart from a few randomized intervention trials, most of the available evidence on the impact of diet on outcomes, such as lung function (FEV1, FVC, FEV1/FVC), symptoms, incidence, prevalence or severity of COPD, and its progression over time, largely comes from observational studies, either cross-sectional or, to a lesser extent, longitudinally in both the general population and at-risk or diseased subjects. The strength of some studies is the use of objective measures of lung function that limit the bias arising from self-reported or physician-diagnosed disease: Post-bronchodilator spirometry is the gold standard for the diagnosis of COPD, minimizing misclassification.

Assessment of dietary intake usually included a 24 h recall and food-frequency questionnaire, both with inherent limitations, including the poor measurement of usual intake due to daily variation in food intake (mostly for 24 h recall), the semiquantitative nature of the assessment, the measurement error, the variation in diet definitions, and the lack of generalizability of study findings among different populations [12]. To estimate the independent association of diet with lung function and COPD, in most studies, the confounding bias is tackled by performing the adjustment for multiple confounding factors known to influence pulmonary function or dietary behavior, including age, gender, BMI, physical activity, intake of other foods or nutrients, energy intake, educational level, and most importantly, tobacco exposure. Sex differences in susceptibility to COPD have been increasingly recognized, with evidence that women are at a greater risk of smoking-induced lung function impairment [28] and poorer health status for the same level of tobacco exposure compared to men [29], and that gender differences may also extend to different food choices [30]. Furthermore, the increased tobacco use recently registered in women likely contributes to the epidemic of COPD in women and influences interpretation of study results. Notably, smokers tend to follow an unhealthy diet compared to ex-smokers [31] and have a higher level of oxidative stress, which is targetable by diet. Moreover, a healthy diet may be associated with other beneficial lifestyles (e.g., higher level of physical activity, higher education, lower BMI, less smoking). Even after adjustment, residual confounding of dietary associations still remains possible and contributes to some inconsistencies across studies.

Many studies have focused on the effects of individual foods or nutrients in relation to respiratory outcomes. However, this information may not completely capture the overall effect of diet on respiratory health nor reflect real life conditions where foods or nutrients are eaten in multiple combinations [32]. Moreover, nutrient and food intakes are closely correlated in the diet, so it can be difficult to disentangle their independent effects. As an example, the lack of benefit of vitamin supplementation on lung function and hospitalization for COPD [33] may be explained, at least in part, by the fact that antioxidant regimens could be effective when adopted in the form of dietary patterns rather than individual nutrients. Dietary pattern analysis captures the quantities, proportions, variety, or combination of different foods and beverages in the diets and provides a framework to evaluate the health effects of the whole diet. This may increase the ability to highlight a stronger impact due to the cumulative effects of many features of the diet and to assess the interaction among synergistic components. This comprehensive approach is emphasized by prevailing dietary guidelines and has been used in several clinical settings, including CVD, cancer, and type 2 diabetes [12].

It should be acknowledged, however, that the evaluation of overall dietary patterns could mask the effects of individual foods or nutrients and disregard potential effects of foods or nutrients not included as components of the pattern. Therefore, the best option could be to complement and integrate data on eating patterns with those of individual components as much as possible in the same study population.

Numerous different combinations of foods and nutrients may be potentially investigated as patterns of food intake, and approaches to rank and quantify adherence of study participants to these patterns have been developed to evaluate their association with disease risk. These approaches [12] include: (1) a priori-defined (hypothesis-driven) indices or scores designed to capture specific dietary patterns defined a priori on the basis of scientific evidence on the relation between food and nutrient intakes and health outcomes; these scores also allow measuring conformity to nutritional requirements and dietary guidelines, with the drawback of considerable variation in the composition of patterns across studies; and (2) data-driven (exploratory) statistical methods (cluster analysis, principal component and factor analysis, and reduced rank regression) to derive existing major patterns of food intake, with the limitation of being specific to the population investigated.

## 5. Oxidant–Antioxidant Imbalance and Diet Quality in COPD

Oxidative stress and associated inflammation in the lung and in the circulation in response to exposure to air pollution, tobacco smoke, infection, or potentially obesity are leading pathogenic processes in COPD. Compared to healthy controls, patients with COPD tend to have increased systemic and airway oxidative damage markers (relative to DNA, lipid, and proteins) [34], coupled to altered antioxidant defense, as evidenced by marked reduction in both plasma antioxidant capacity and soluble and enzymatic antioxidants levels [34,35,36]. Moreover, oxidative stress persists long after smoking cessation as a result of continuous production of pro-oxidants [37]. Low serum antioxidant vitamin levels appeared to increase the risk of obstructive airways diseases associated to smoking exposure [38]. In accordance, higher levels of oxidative markers in COPD were correlated with decreased lung function [39,40,41], while higher serum levels of antioxidant enzymes (catalase, superoxide dismutase, glutathione peroxidase) [40,41], as well as of soluble antioxidants (vitamins, carotenoids, etc.) [35,42,43], were positively associated with lung function. Therefore, it can be hypothesized that targeting oxidative stress with antioxidants or boosting endogenous levels of antioxidants might be beneficial in COPD.

Diet may contribute to antioxidant/oxidant and inflammatory status in COPD. Compared to healthy controls, COPD subjects have diets with lower fruit and vegetable intake [44] and with poorer antioxidant content, which was correlated with impaired lung function and risk of having COPD [35,36]. Moreover, lower energy intake (accompanied by elevated resting energy expenditure), unbalanced intake of macronutrients (e.g., low proteins), and defective intake of several micronutrients (minerals and vitamins, e.g., iron, calcium, potassium, zinc, folate, vitamin B6, retinol, niacin) have been documented in COPD patients compared to healthy controls [45], mostly in the presence of obesity [46], suggesting an increased risk of malnutrition and related adverse consequences in COPD.

The poor diet quality and the nutrient deficiencies in COPD, which are related to disease-specific factors such as symptoms (e.g., dyspnea, fatigue, anxiety, depression, anorexia, periodontal disease, loss of taste, poor dentition, dysphagia, poor chewing and swallowing ability) or social problems (e.g., living or eating alone, or poverty) [47], require improvement through dietary intervention to satisfy nutritional requirements and even to supplement further protective factors able to counteract disease pathogenesis. The inflammatory/oxidative status in COPD and the associated procatabolic state contributing to weight loss and muscle wasting in severe COPD represent further possible targets for nutritional intervention.

## 6. Individual Foods and Nutrients, Lung Function, and COPD

### 6.1. Role of Antioxidant and Anti-Inflammatory Foods: Fruits and Vegetables

The dietary quality and the nutritional status of COPD patients as well as the oxidative–inflammatory pathogenic basis of COPD provided the rationale to verify the respiratory effects of antioxidant and anti-inflammatory dietary components. Consistent epidemiologic evidence from cross-sectional [36,38,48,49,50,51] and longitudinal studies [42,52,53,54,55] reported potential beneficial effects of a high intake of antioxidant nutrients (vitamins and nonvitamins) and of foods rich in antioxidants, mostly fresh, hard fruits and, to a lesser extent, vegetables, on lung function and COPD symptoms [35,36,49,50,51], decline in lung function [42,49,56], incidence of COPD [52,54,55], and mortality [53].

Two recent large population-based prospective studies in Swedish men [54] and women [55] confirmed the inverse and independent association between high long-term consumption of fruits (in both men and women) and vegetables (only in men) and incidence of COPD (35% lower risk in men, *p* for trend <0.0001 [54]; 37% lower risk in women, *p* for trend <0.0001 [55]). These beneficial dietary associations were particularly evident among smokers. Specifically, in the cohort of men (with higher smoking intensity than women), the protective effect was restricted to current and ex-smokers (40% lower risk, *p* for trend <0.0001, and 34% lower risk, *p* for trend = 0.001, respectively), mostly benefiting from dietary antioxidants, probably as a result of increased oxidative stress level due to smoking compared with never smoking, and the continued oxidative burden even after smoking cessation [54]. Regarding individual food items, intakes of apples, pears, peppers, and green leafy vegetables were negatively associated with the risk of COPD [54].

Few randomized dietary intervention trials have been conducted. In a small 12-week randomized trial including moderate-to-severe COPD patients complying with an intervention to increase fruit intake, no improvement in airways or systemic inflammation and oxidative stress markers was observed, although the follow-up might have been too short to observe any significant effect [57]. In COPD patients, 1-week supplementation with beetroot juice, a dietary source of nitrates that improves mitochondrial respiration and energy production via nitric oxide formation, increased plasma nitrate levels and decreased diastolic blood pressure (mean difference 4.6 mmHg, 95% CI: 0.1, 9.1, *p* < 0.05) without any effect on walking capacity, physical activity level, or oxygen consumption of submaximal exercise [58]. However, another randomized trial reported that COPD patients following a diet rich in fruits and vegetables (>1 portion/day) showed an annual increase in FEV1 compared with the control group following a free diet over 3 years (*p* = 0.03), after adjustment for physical activity, alcohol intake, co-morbidities, and exacerbation frequency [59].

Collectively, these observations suggest fruit and vegetable consumption as an important determinant of pulmonary function and COPD risk. It should be noted that fresh fruit intake may be one component of an overall healthier lifestyle, including less smoking, more physical activity, low consumption of Western foods (e.g., meat), and increased consumption of vegetables [54,55,59], and other not checked nutrients may mediate the observed beneficial effects. Furthermore, assessment of blood, urine or exhaled breath condensate biomarkers of endogenous oxidative stress is generally lacking in most longitudinal studies, thus limiting the possibility to more accurately select subjects more susceptible to antioxidant dietary regimens and to appraise the antioxidant efficacy of tested foods over time.

### 6.2. Vitamin and Nonvitamin Antioxidants

Plausible mechanisms underlying fruit and vegetable protective effects include their antioxidant and anti-inflammatory activities, as suggested by the epidemiologic association observed between fruit and vegetable consumption and lower markers of oxidative stress and inflammation, and higher levels of antioxidant markers [60,61]. Fruits’ and vegetables’ beneficial effects on respiratory function may be partially contributed by their high content in vitamin and nonvitamin antioxidants. Accordingly, higher dietary intakes of vitamin C, a hydrophilic antioxidant, were associated with higher levels of FEV1 [35,62] and with a lower rate of decline in FEV1 after a 9-year follow-up period [62]. Other studies did not confirm a significant effect of vitamin C dietary intake on lung function (FEV1), its longitudinal decline [49], COPD incidence [52] or mortality [53]. Although not consistently [62], a protective role has also been credited to other vitamins such as vitamin E or tocopherol, a lipid soluble antioxidant acting in synergy with vitamin C and able of breaking lipid peroxidation chain reaction and protecting the lung against oxidative damage [36,53]. Lower serum vitamin E levels have been observed in COPD during exacerbation compared to stable condition [63]. Randomized trials of vitamin E supplementation in clinical populations have, however, reported mixed results, including both protective [64] and no effects [33] on the risk of developing COPD.

Butland et al. [49] found a positive cross-sectional association between higher consumption of hard fruits, such as apples (5 or more apples per week) and lung function (FEV1) (138 mL higher FEV1 for those eating 5 or more apples per week compared with nonconsumers, 95% CI: 58.1, 218.1, *p* for trend <0.001), more strongly than soft or citrus fruits and independent of vitamin E and vitamin C intakes. Similarly, Miedema et al. [52] found a stronger inverse association with 25-year incidence of COPD for solid fruits (apples, pears) than for other types of fruits. Other nonvitamin dietary components may therefore exert protective effects. These include the fat-soluble antioxidant carotenoids (lycopene, lutein, zeaxanthin, and the provitamin A carotenoids α-carotene, β-carotene, and β-cryptoxanthin), whose serum and dietary levels have been positively correlated to lung function indicators (FEV1, FVC) [35,42,65]. However, long-term supplementation with β-carotene or α-tocopherol failed to reduce COPD symptoms in a large cohort of male smokers randomized into the α-tocopherol and β-carotene Cancer Prevention (ATBC) Study [66]. Notably, strong evidence from randomized controlled trials conducted in heavy smokers and asbestos-exposed workers, i.e., the β-carotene and retinol efficacy trial (CARET) [67] and the abovementioned ATBC study [68], showed that high-dose β-carotene supplements may increase the risk of lung cancer and of death from lung cancer, CVD, and any cause. Contrarily, this harmful effect was not observed among healthy male physicians in the Physicians’ Health Study in the USA [69]. An interaction of carotenoids with cigarette smoking has been proposed to explain the shifting of carotenoid antioxidant potential into a pro-oxidant detrimental effect on the lung: β-carotene can easily form oxidation products with pro-oxidant effects, especially at high concentrations in the oxidative environment of the smoker’s lung characterized by increased cell oxidative stress and decreased antioxidant defense [70]. Against this background, according to the 2018 report of the World Cancer Research Fund/American Institute for Cancer Research (https://www.wcrf.org/dietandcancer), β-carotene supplements (and dietary supplements in general) are not recommended for cancer prevention (especially in smokers), while intake of natural micronutrients through diet is advisable. Therefore, caution should be taken, especially in smokers, when considering dietary supplementation with β-carotene.

Other potentially protective dietary factors include polyphenols, the most abundant antioxidants in human diets naturally present in plant foods, and exhibiting potent anti-inflammatory properties. Polyphenols, including phenolic acids, flavonoids (flavonols, flavones, isoflavones, flavanones, flavanols, and anthocyanidins), stilbenes (resveratrol, etc.), lignans, and secoiridoids, have been reportedly associated with prevention of chronic diseases, including CV and neurodegenerative diseases and cancer, and with the promotion of healthy aging [71]. Beneficial effects on respiratory function have been reported for the flavonoid class of polyphenols: In an earlier Dutch study, the intake of catechins was positively associated with FEV1 (mean difference in FEV1 comparing high vs. low intake of catechins = 130 mL, 95% CI: 101–159, *p* < 0.05) and negatively associated with all three COPD symptoms (odds ratio (OR) of phlegm, breathlessness and cough, comparing high vs. low intake of catechins = 0.60–0.72, *p* < 0.001) [50]; concordantly, slower longitudinal decline in lung function was observed with higher intake of anthocyanidins in US elderly men [72], and a beneficial association of dietary intakes of isoflavones as well as of soy, which is a rich food source of isoflavones, with lung function and COPD prevalence was also observed in Japanese adults [73,74]. In a recent randomized trial in COPD patients, supplementation with flavonoids in the form of oligomeric pro-anthocyanidins extracted from grape seeds was effective in improving oxidative stress and lipid profile, but not lung function parameters after 8 weeks [75]. More recent observational findings in a Mediterranean population confirmed protective effects of the intake of various polyphenol classes on pulmonary function parameters [76]. In a cross-sectional study in 267 Spanish COPD patients, dietary intakes of vitamin E as well as vegetables and olive oil, rich in vitamin E and polyphenols, have been shown to be inversely correlated with serum markers of oxidative stress, especially in current smokers [77]. In an Asian population-based cross sectional study, a diet rich in the potent antioxidant and anti-inflammatory turmeric-derived polyphenol curcumin was significantly and independently associated with improved lung function measures, and smokers with the highest curcumins intake had levels of lung function greater than smokers not consuming curcumins and similar to those of nonsmokers, supporting the antioxidant and anti-inflammatory dietary hypothesis [78].

### 6.3. Minerals

Among micronutrients, cross-sectional studies have found deficient intake of some minerals in COPD patients. Indeed, dietary intakes and serum levels of calcium, magnesium, and selenium were found to be below the recommended values in older, underweight patients with severe COPD [79]. Lower intakes of calcium and zinc were observed in elderly COPD patients compared with non-COPD subjects [45]. Some minerals have been studied in relation to lung function and COPD risk and symptoms. A case-control study in Japanese adults found a positive association between intake of calcium, phosphorus, iron, potassium, and selenium and lung function measures (e.g., FEV1), and an inverse association between dietary calcium intake and COPD risk (35% reduction) [80]. FEV1 was independently and positively associated with serum levels of selenium, normalized calcium, chloride, and iron, and was inversely related to potassium and sodium in the general population [43]. Other cross-sectional studies confirmed the association between serum levels of selenium as well as copper and higher lung function [81]. A randomized placebo-controlled trial reported that selenium supplementation (200 µg/d L-selenomethionine), either alone or in combination with vitamin E (400 IU/d all rac-α-tocopheryl acetate), did not affect decline in FEV1 or FEF25–75, a marker of airflow, but attenuated decline in FEF25–75 (by 59 mL/second/year) in current smokers, who may benefit most from selenium supplementation due to its potent antioxidant properties linked to the glutathione peroxidase activity [82].

Early population-based studies reported a strong association between magnesium intake and lung function, airway hyper-responsiveness, and wheeze [83], although this result was not consistently found [49]. More recently, in a general UK population cohort intake of magnesium was cross-sectionally related to higher FEV1 (a 100 mg/day higher magnesium intake was associated with a 52.9 ml higher FEV1 (95% CI, 9.6–96.2)), but no relationship between intake of magnesium and longitudinal decline in FEV1 was seen [62]. Similar results were recently obtained by Leng et al. [84] in New Mexico white smokers. Magnesium may play a beneficial role in respiratory function and COPD, through its protective effects against inflammation and bronchoconstriction [85]. Although the limited evidence suggests protective effects of some minerals on lung function and COPD, mostly for those endowed with antioxidant and anti-inflammatory properties, further prospective studies are warranted.

### 6.4. Wholegrains and Fibers

Among dietary factors largely investigated, mostly in relation to CVD and cancer, research has also focused on wholegrains. Observational studies reported an independent beneficial effect of a high wholegrain intake on lung function [51,86], and against mortality from chronic respiratory disease [87]. Wholegrains are rich in phenolic acids, flavonoids, phytic acid, vitamin E, selenium, and essential fatty acids, which may additively or synergistically contribute to wholegrain documented beneficial effect on respiratory as well as nonrespiratory diseases.

Part of the protective action of wholegrains as well as of fruits and vegetables is attributable to the antioxidant and anti-inflammatory properties of their fiber content [88]. Indeed, epidemiological data indicated that fiber intake is associated with lower serum levels of C-reactive protein and cytokines (IL-6, TNF-α) and higher level of adiponectin, an insulin-sensitizing adipocytokine with anti-inflammatory properties [89]. In line with these beneficial properties, cross-sectional and longitudinal studies found a negative and independent association between total fiber intake and lung function decline, and COPD incidence and prevalence [90,91,92]. Indeed, higher dietary intake of total fiber reduced by about 40% the risk of COPD in large prospective studies [91,92]. Considering fiber types (cereal, fruit, vegetable), the beneficial association was observed mostly for cereal fiber intake mainly in current smokers and ex-smokers, but evidence exists also for fruit and vegetable fiber intake [91,92].

### 6.5. Alcohol and Wine

Other significant associations with respiratory health have been documented in the general population for intake of alcohol and wine. Previous epidemiologic studies found that subjects with low alcohol consumption (1–30 g/day) had higher levels of FEV1, lower prevalence of COPD symptoms [51], and a decreased risk of COPD compared to nonconsumers [52]. By contrast, heavy alcohol intake, as assessed by both dietary and serum biomarker measurements, was shown to have negative effects on lung function, additive to that of smoking [93]. Among the different alcohol sources, only wine intake (>7.4 g/day) was found to be positively associated with FEV1 in the general population [94], as well as with a lower risk of airway obstruction, defined as an abnormally low FEV1/FVC ratio, predominantly in smokers [95]. Beyond direct protective effects of alcohol as previously reported [96], putative candidates accounting for the observed beneficial effect of wine are flavonoids [50], as well as the stilbene resveratrol [95], both associated with improved measures of lung function. Congruently, resveratrol has been reported to exert anti-inflammatory properties in airway epithelial cells [97], alveolar macrophages derived from COPD patients [98], and airways smooth muscle cells [99], and the flavonol quercetin has been shown to attenuate rhinovirus-induced lung inflammation and emphysema progression in a mice model of COPD [100].

Interestingly, the independent beneficial effects of a favorable intake of fruits (>180 g/day), wholegrains (>45 g/day), and alcohol (1–30 g/day) on FEV1 and COPD symptoms were additive (favorable vs. unfavorable intake, 139 mL higher FEV1 and COPD symptoms prevalence OR = 0.44, *p* < 0.001) [51], suggesting important interaction among nutrients and food groups. Moreover, findings from the ECLIPSE study in COPD subjects demonstrated that recent consumption of “healthy” foods, such as fruits (grapefruit and bananas), fish, tea, dairy products, and alcohol, was associated with higher lung function and less decline over time, less emphysema and emphysema progression, greater 6-minute walk and St. George’s Respiratory Questionnaire (SGRQ) scores, and lower levels of inflammatory markers (C-reactive protein, white blood cells, surfactant protein D, total neutrophils) [101]. These data extend the role for dietary intakes to phenotypic features of COPD patients.

### 6.6. Vitamin D

Limited evidence also supports a direct correlation between vitamin D levels, which mainly depend on sun exposure in addition to diet, and lung function, COPD incidence, symptoms, severity and progression [102,103,104]. Genetic variants in the vitamin D-binding protein associated with lower plasma vitamin D levels have also been linked to COPD risk [105]. Mechanistic studies support a role for vitamin D other than calcemic effects and in particular in normal growth and development of the lung as well as in immune responses and COPD progression. Vitamin D supplementation trials to prevent COPD exacerbation reported conflicting results but, collectively, pointed to a benefit only in patients with low baseline vitamin D levels (i.e., levels of active metabolite 25-hydroxyvitamin D <25 nmol/L) [106]. Although further studies are needed, taking into account the highly prevalent osteoporosis and risk of falls in COPD patients and also the supposed beneficial effects of vitamin D beyond bone health, screening for vitamin D deficiency (25-hydroxyvitamin D <50 nmol/L) may be important in COPD patients.

### 6.7. Coffee and Its Components

Given its widespread consumption, interest has been growing around the potential role of coffee in respiratory health. Findings from literature reviews point to an association between regular (not decaffeinated) coffee intake and improved lung function and reduced mortality from respiratory disease, but not COPD [107], with contributory roles for its constituents, caffeine (bronchodilator, anti-inflammatory) and polyphenols (antioxidant, anti-inflammatory). Smoking is a major confounder in these studies because it may accelerate the hepatic metabolism and clearance of caffeine or may dilute or dampen the beneficial effects of coffee through its potent pro-oxidant and pro-inflammatory action [107].

### 6.8. Role of Fish and n-3 Polyunsaturated Fatty Acids

α-Linolenic acid (ALA, C18:3) and its long-chain derivatives eicosapentaenoic acid (EPA, C20:5) and docosahexaenoic acid (DHA, C22:6) are polyunsaturated fatty acids (PUFA) of the *n-3* (omega-3) family. Due to the low efficiency of endogenous synthesis from precursors, they are considered nutritionally essential and depend on exogenous source, mainly seafood (fatty fish). *n*-3 PUFAs and fish display potent anti-inflammatory properties with beneficial effects and, in most cases, clinical applications in several chronic inflammatory diseases, including CVD, cancer, rheumatoid arthritis, and diabetes [108]. Opposite effects have been described for *n*-6 PUFAs, including linoleic acid (LA, C18:2) and its long-chain derivative arachidonic acid (AA, C20:4), mainly found in vegetable oils (soybean, corn, and sunflower oils), grain-fed animals, dairy, and eggs. Indeed, metabolism of long-chain *n-6* PUFA produces eicosanoids (such as thromboxane(TX) A2, prostaglandin(PG) E2 and leukotriene(LT) B4) which are more potent mediators of inflammation, thrombosis, and vaso- and bronco-constriction than similar products derived from *n*-3 PUFAs (PGs of the 3-series and LTs of the 5-series) [109]. Some EPA and DHA metabolites via cytochrome P450 enzymes, which are highly expressed in the lungs, are potent vasodilators and bronchodilators and show anti-inflammatory properties. Other metabolites of long-chain *n*-3 PUFAs include the inflammation-resolving eicosanoids resolvins and protectins, which act to remove inflammatory mediators and promote healing.

Increasing the content of *n*-3 PUFA in the diet causes a partial substitution of the *n*-6 PUFA in the cell membranes and competition for the metabolizing enzymes, thus favoring the synthesis of generally less biologically active eicosanoids. In addition to lipid metabolites, *n*-3 PUFA anti-inflammatory mechanisms also include the direct modulation of inflammatory gene expression (adhesion molecules, cytokines, matrix degrading enzymes, cyclooxygenase-2) via the regulation of nuclear transcription factors, mainly the oxidative stress-sensitive pro-inflammatory NF-κB [109], which has also been involved in the pathogenesis of lung inflammation [24].

The secular trend of dietary shift to a more Westernized pattern and the following increased consumption of *n*-6 PUFAs and decreased consumption of *n*-3 PUFAs is thought to have contributed to the rise in chronic inflammatory disease [108]. Given the role of inflammation in COPD and the reported benefits of long-chain *n*-3 PUFAs in many inflammatory diseases, studies have been conducted to verify the ability of dietary PUFAs to also modulate COPD (prevalence, severity, and health outcomes). However, as underlined in a recent systematic review [110], data are conflicting. Some earlier observational studies suggest benefits from increased consumption of *n*-3 PUFA-rich foods, in particular fatty fish, on respiratory function and COPD symptoms, mainly among smokers, but none of the studies adjusted for other dietary intakes [111,112,113]. Contrarily, in a 25-year prospective study conducted in the Netherlands, the intake of *n*-6 PUFA was positively related to the incidence of chronic lung diseases (defined as chronic productive cough, chronic bronchitis, emphysema, and asthma), but no relation between *n*-3 PUFA intake and the incidence of chronic nonspecific lung disease was observed [52]. Concordantly, other studies did not find any independent beneficial association between fish intake and FEV1, COPD symptoms [51] or mortality [53]. A large population-based cross-sectional study found that higher intake of individual *n-6* PUFAs was associated with lower FEV1 (reduction in FEV1 between the highest vs. lowest quintile of intake = 54.5 mL, 95% CI: –81.6, –27.4, *p* < 0.0001), particularly in smokers, and with increased risk of COPD, while no association between individual *n*-3 PUFAs intake and FEV1 or COPD symptoms was seen [114].

More recently, in two large US cohorts, while an initial analysis showed that higher intake of fish (≥4 servings/week), but not PUFAs, was associated with lower risk of newly diagnosed COPD, after adjustment for overall dietary pattern, this association lost significance, suggesting that potential benefits of fish might be evident within the whole diet [115]. Interestingly, fish intake could reduce the risk of COPD when intake of plant sources of *n*–3 PUFAs is high [115], suggesting that a healthy diet including fish as well as vegetable sources of *n*-3 PUFAs may be more beneficial for COPD than isolated food or nutrient.

Evidence exists that anti-inflammatory actions of *n*-3 PUFA may extend and be relevant to COPD pathogenesis. In an in vitro study, shifting the PUFA supply from AA to DHA significantly reduced the release of pro-inflammatory cytokines (TNF-α, IL-6, and IL-8) and increased the release of anti-inflammatory cytokine (IL-10) from human alveolar cells after endotoxin challenge [116]. Resolvin D1 (derived from DHA) has been reported to inhibit cigarette smoke-induced pro-inflammatory response in human lung cells in vitro and in a mouse model of acute cigarette smoke-induced lung inflammation by selectively activating specific anti-inflammatory pathways, including the inhibition of neutrophilic inflammation and the activation of a subset of anti-inflammatory, pro-resolving macrophages [117]. In stable COPD patients, higher circulating inflammatory markers (IL-6, C-reactive protein) were associated with higher dietary intake of *n-6* PUFAs (for IL-6, OR = 1.96, *p* = 0.034; for CRP, OR = 1.95, *p* = 0.039), while lower plasma levels of the cytokine TNF-α were related to *n*-3 PUFAs intake (OR = 0.46, *p* = 0.049) [118]. Results from feeding trials assessed health outcomes in COPD patients. An 8-week supplementation with *n*-3 PUFA (1200 mg ALA, 700 mg EPA, and 340 mg DHA) in patients with moderate-to-severe COPD reversed muscle wasting and improved the functional capacity compared with placebo, without any effect on FEV1 or systemic inflammatory markers (CRP, IL-6, and TNF-α) [119]. Further studies, especially randomized controlled trials, are therefore needed to appraise the relationships between intake of long-chain *n*-3 PUFA and/or fish and COPD.

### 6.9. Foods with Potential Deleterious Effects on Lung Function and COPD

Among potential deleterious foods, a statistically significant inverse association between frequent consumption of cured (bacon, hot dogs, and processed meats) and red meats and pulmonary function has been reported, in agreement with evidence of detrimental effects in other nonrespiratory diseases, including CAD, diabetes, and cancer [120,121], and all-cause mortality [122]. Increased intake of cured meats was independently associated with an obstructive pattern of spirometry in a cross-sectional analysis in the third National Health and Nutrition Examination Survey [123] and with an increased risk of newly diagnosed COPD in both men and women in US prospective cohorts, independent of Western dietary pattern (highly loaded with red meat) or other associated dietary intakes (refined grains, desserts, etc.) [124,125]. Importantly, more recent large Swedish population-based prospective studies confirmed this detrimental effect for both baseline and long-term consumption of processed (not unprocessed) red meat [126,127]. Another study found that cured meat intake increased the risk of COPD readmission [128]. Collectively, as summarized in a recent meta-analysis, available evidence indicated a 40% increased risk of COPD with higher consumption of processed red meat (>75–785.5 g/week) [129].

These data suggest that health-promoting activities should include specific advice on lowering red/processed meat consumption. It would be important to confirm these results in those populations experiencing nutrition transition with an increased consumption of Westernized foods, including processed meats.

In addition to the high content in cholesterol and saturated fatty acids, drawbacks of processed red meat include the presence of nitrites, which are added to processed meat during the manufacturing process as a preservative, antimicrobial, and color fixative. Nitrites generate reactive nitrogen species, such as peroxynitrite, with the subsequent nitrosative stress that can contribute to, and amplify, inflammatory processes in the airways and lung parenchyma, causing DNA damage, inhibition of mitochondrial respiration, and cell dysfunction. Moreover, tyrosine nitration in connective tissue proteins, including collagen and elastin, can alter their function. Higher levels of nitrotyrosine have been observed in subjects with COPD and were correlated to disease severity [130]. Accordingly, in animal models, chronic exposure to nitrite caused emphysema-like pathological changes in the lungs [131]. Nitrites are also byproducts of tobacco smoke; thus, nitrite generation may be one of the mechanisms by which tobacco smoke causes COPD. Congruently, the combination of smoking and higher cured meat consumption is indeed associated with the highest risk of newly diagnosed COPD [125]. Cured meats also contain a high amount of sodium that may increase bronchial hyper-reactivity and may elicit inflammation [132]. Sodium dietary intake has been reported to be higher in COPD patients compared to healthy controls and to be associated with lower lung function [80].

Meat is also an important source of saturated fatty acids (SFAs), which can trigger inflammation, also in the airways [133], and have been associated with both impaired lung function [134] and an elevated risk of coronary heart disease and metabolic diseases [135]. This risk seems to be mainly attributable to medium and long chain SFAs (C14:0–C18:0) highly present in meat compared to other animal sources such as dairy products. By contrast, increased intake of low-fat dairy products [136] as well as of short and medium chain SFAs, as assessed by 24 h recall [137], may exert protective effects on lung function, possibly through their anti-inflammatory action.

An important feature of the Western lifestyle and diet is the consumption of foods with high glycemic index, such as refined grains, desserts, sweets, and sweetened beverages. In addition to increasing the risk of obesity, hyperglycemia may trigger oxidative stress-related inflammatory responses [138], is associated with impaired lung function [139] and poor COPD outcomes [140], and may promote pulmonary infection, at least in part, by an effect on airway glucose concentrations [141]. Part of the detrimental effects of hyperglycemia is mediated by the formation of advanced glycation end-products (AGEs), which are elevated in lung tissues of COPD patients and are known to be associated with lung inflammation and pathophysiology [142]. Compared to no consumption, high levels of soft drink consumption (>0.5 L/day, sweetened or not), an important component of the Western lifestyle and diet, were associated with a higher prevalence of COPD (OR = 1.79, 95% CI: 1.32, 2.43, *p* < 0.001) and asthma (OR = 1.26, 95% CI: 1.01, 1.58, *p* = 0.014), in an additive manner with smoking [143]. Moreover, consumption of excess fructose-sweetened soft drink (>5 times/week) was significantly correlated to chronic bronchitis in US adults (OR = 1.80, 95% CI: 1.01, 3.20, *p* = 0.047) [144], as well as to pediatric asthma [145], possibly due to the formation of AGEs from the interaction between unabsorbed free fructose and dietary proteins in the gastrointestinal tract. These results clearly emphasize the public health implication of interventions targeting modern unhealthy lifestyle habits.

## 7. Dietary Patterns, Lung Function, and COPD

Dietary patterns have been widely investigated in relation to cancer, CVD or diabetes [12], but limited data are available on their association with respiratory outcomes with relevance to COPD. As shown in a recent meta-analysis [14], most studies were performed in Europe and North America, limiting the generalizability of study findings, and were observational in design. Overall, the evidence concordantly indicated that the pattern of dietary intake is an important factor in the pathogenesis and prevention of COPD and provided support for specific dietary modifications as a clinically relevant tool to promote lung health. Moreover, examination of dietary patterns complements the evaluation of the effects of individual food and nutrient intake on COPD. Table 1 summarizes findings from main epidemiological studies addressing the relation between diet and lung function, COPD risk, symptoms, and progression.

### 7.1. Data-Driven Dietary Patterns and COPD

A cohort study in Chinese Singaporeans found that the meat–dim sum dietary pattern (red meat, preserved foods, rice, noodles, deep-fried foods) was associated with an increased incident cough with phlegm (odds ratio (OR) = 1.43 comparing fourth to first quartile, *p* for trend = 0.02) [146], indicating a deleterious effect of a diet rich in meat, starchy foods, and high-fat dairy products on respiratory symptoms. Two prospective studies in US health professionals identified two distinct major dietary patterns, the “prudent pattern”, loaded by a high intake of fruits and vegetables, oily fish, poultry, wholegrain products, and low-fat dairy products, and the “Western pattern”, characterized by a high consumption of refined grains, cured and red meats, desserts, French fries, and high-fat dairy products [147,148]. Both studies consistently found that the “prudent” pattern was negatively and the Western pattern positively associated with the risk of self-reported newly diagnosed COPD in women [148] and men [147] after adjustment for several potential confounders, including measures of tobacco exposures. In contrast with findings for COPD, the dietary patterns were not associated with the risk of adult-onset asthma. Notably, the effect of each dietary pattern was stronger in men than in women [147,148]. For the prudent pattern, the relative risk (RR) for highest vs. lowest quintile was 0.50 (*p* for trend = 0.02) in the men cohort [147], and 0.75 (*p* for trend = 0.02) in the women cohort [148]. For the Western pattern, the RR for highest vs. lowest quintile was 4.56 (*p* for trend <0.001) in the men cohort [147], and 1.31 (*p* for trend = 0.02) in the women cohort [148]. As several individual foods of the “prudent” or the “Western” diet might be related to COPD, as discussed in previous paragraphs, the “prudent” and the “Western” diet patterns reflect the possible combinatory effects of these diverse but highly correlated foods.

Cross-sectional studies confirmed the associations between dietary patterns and respiratory symptoms, lung function, and COPD. A study in the UK population observed that a similar “prudent” dietary pattern was positively associated with lung function (FEV1) in males and females (difference in mean FEV1 between highest vs. lowest quintiles of pattern score = 180 mL in men, *p* for trend <0.001, and 80 mL in females, *p* for trend = 0.008), and negatively associated with COPD prevalence in males (54% reduction, *p* for trend = 0.012) [149]. Associations in males were stronger among smokers than nonsmokers [149]. In this study, the second dietary pattern identified in the study subjects, i.e., the “traditional” pattern, was similar to the unhealthy Western dietary pattern of other studies [147,148], but here, it was not associated with any negative outcome, probably because of its relatively high fish and vegetables content. A “prudent” diet was confirmed to be associated positively with FEV1 and negatively with COPD prevalence, not significantly in a large sample (*n* = 2,178) of Swiss adults (changes in FEV1 per unit increment in the dietary pattern score = 23 mL, *p* = 0.08; COPD prevalence OR = 0.90, *p* = 0.21) [150], as well as in a significant manner in a US population-based study (COPD prevalence OR = 0.82, *p* for trend = 0.007) where protective effects by the prudent pattern were also observed on respiratory symptoms (cough), whereas a Western diet was associated with higher prevalence of COPD (OR = 1.62, *p* for trend <0.001), worse respiratory symptoms, and lower lung function [151].

In a population of around 12,000 adults from the Netherlands, McKeever et al. [152] identified three major dietary patterns, the “cosmopolitan pattern” (higher intakes of vegetables, fish, chicken, wine, and lower intakes of high-fat dairy products, added fat, added sugar, and potato), the “traditional pattern” (higher intakes of red meat, processed meat, potato, boiled vegetables, added fat, coffee, and beer and lower intakes of soy products, low-fat dairy products, tea, breakfast cereal, brown rice, pizza, juice, and fruit), and the “refined food dietary pattern” (higher intakes of mayonnaise, salty snacks, candy, high-sugar beverages, French fries, white bread, and pizza and lower intake of boiled vegetables, wholegrains, fruit, and cheese). These dietary patterns were analyzed for their relation to lung function (FEV1) and symptoms of COPD as well as to longitudinal change in FEV1. When nutrient intake associated with the diets was analyzed, the “cosmopolitan” diet was positively correlated with intake of alcohol, vitamin C, and beta-carotene, the “traditional” diet was positively associated with alcohol and total fat intake, and negatively with carbohydrate intake, and the “refined food” diet was negatively associated with magnesium, fiber, and vitamin C intake [152]. In the cross-sectional analysis, the “traditional” diet was associated with a lower lung function (−94.4 mL, *p* for trend <0.001) and an higher prevalence of COPD (OR = 1.6, *p* for trend = 0.001), while the “cosmopolitan” diet was associated with a small increased prevalence of asthma and wheeze. None of the dietary patterns were associated with a decline in lung function over 5 years, although a higher intake of refined foods was associated with a greater decline in lung function (−48.5 mL, *p* for trend = 0.11) [152].

Accordingly, in a Spanish population of adult smokers without respiratory diseases, three major dietary patterns were derived: alcohol-consumption pattern (loaded by intake of wine, beer, and/or distilled drinks), Westernized pattern (loaded by high consumption of cured and red meats, dairy products, and sugary drinks, desserts and sweets, and low in fruits, vegetables, legumes, and fish), and Mediterranean-like pattern (loaded by high intake of poultry, eggs, fish, vegetables, legumes, potatoes, dairy desserts, fruits, nuts, and dried fruit) [153]. When the prevalence of impaired lung function (as determined by spirometry) across tertiles of dietary patterns was analyzed, impaired lung function was observed in all participants with an alcohol consumption pattern (OR = 4.56, *p* for trend = 0.005) especially in women (OR = 11.47, *p* for trend = 0.003), and in women with the Westernized pattern (OR = 5.62, *p* for trend = 0.031). By contrast, the Mediterranean-like dietary pattern was not significantly associated with impaired lung function, but with a trend for preserved lung function (OR = 0.71, *p* for trend >0.05), suggesting that it may protect lung function against the deleterious effects of smoking [153].

The study by Sorli-Aguilar et al. [153] provides some new information: (1) it restricted the observation to smokers, thus stressing the importance of eating pattern, in addition to smoking cessation, as a possible preventive measure for improving lung health; (2) it provides a first report on the association between a Mediterranean-like diet and lung function. An impressive and unprecedented accrual of high-quality evidence from observational and interventional studies converges to the recognition of the traditional Mediterranean diet as one of the healthiest dietary patterns, being protective against incidence and mortality of major chronic diseases, mainly CVD and cancer [157,158]. However, limited evidence exists for a role in obstructive respiratory diseases and mostly regards asthma [159]. As discussed above, many individual foods and nutrients characteristic of the Mediterranean diet and endowed with anti-inflammatory, antioxidant, and beneficial metabolic properties (fruits, vegetables, seafood, nuts, legumes, vitamins, polyphenols, etc.) have been associated to improved lung function and COPD prevention in several studies. The Mediterranean-like diet pattern described by Sorli-Aguilar et al., the healthiest one compared to the other dietary patterns identified, included key foods of the traditional Mediterranean diet (fruits, vegetables, legumes, wholegrains, nuts, olive oil, fish) [160] and was similar to the “prudent” patterns that have been previously found to protect against impaired lung function and COPD risk [147,148,149]. However, it cannot be strictly defined as a traditional Mediterranean diet because it also included non-Mediterranean diet/unhealthy components, such as red and processed meats, desserts, sweets, and refined grains [153]. This may have diluted or masked the possible positive effect on lung function by other beneficial components. Of course, more investigations (mostly interventional in design) in different populations and countries are needed to confirm Mediterranean diet health benefits in COPD.

### 7.2. Diet Quality Scores and COPD

In addition to data-driven approaches to derive dietary pattern, a priori-defined diet quality scores have also been used to assess and/or confirm the relationship of diet with lung function and risk and outcomes of COPD (Table 1). In order to measure compliance to the Dietary Guidelines for Americans (DGAs) and provide dietary guidance for healthy eating, two dietary indices, the Healthy Eating Index (HEI) [161] and the Alternate Healthy Eating Index (AHEI) (2005 and 2010 editions) [162], a modified version of the HEI, have been developed and used in the US population and subpopulations. Apart from some distinctive features, such as more attention to fat quality, inclusion of moderate alcohol intake, cereal fiber, red-to-white meat ratio, and duration of multivitamin use in the AHEI compared to the original HEI, both scores reflect a dietary pattern characterized by high intakes of wholegrains, PUFAs, nuts, and long-chain *n*-3 fats and low intakes of red/processed meats, refined grains, and sugar sweetened beverages, and have been found to beneficially impact health outcomes. Indeed, the AHEI was inversely associated with incidence and mortality from chronic diseases (CVD, diabetes, and cancer) [163,164]. Using the AHEI-2010 score, a recent several year-long prospective study in participants of the US Nurses’ Health Study (NHS, *n* = 73,000 women) and Health Professionals Follow-up Study (HPFS, *n* = 47,000 men) [154] found that higher AHEI-2010 diet score was associated with a 33% lower risk of newly diagnosed COPD in both men and women, without any effects by smoking status and after adjustment for several confounding factors (multivariable HR for eating the healthiest diet compared to eating the least healthy diet = 0.67, *p* for trend <0.001). This negative association also persisted after excluding participants with cancer and CVD at baseline (multivariable HR for eating the healthiest diet compared with eating the least healthy diet = 0.71, *p* for trend = 0.007), indicating a direct effect of a healthy diet on COPD beyond its association with other chronic diseases. Contrarily, no association was found between AHEI and the risk of adult onset asthma. Although obtained in health professionals with differences in health awareness, socioeconomic status, and smoking behavior compared to the general population, these results extend the relevance of the AHEI-2010 diet score and its main dietary features to COPD. When the association between individual components of the score and the risk of COPD was analyzed, high intake of fruit and wholegrains, and low intake of red and processed meat and sugar sweetened drinks and fruit juice were associated with lower risk of COPD [154], confirming some previous findings about the respiratory benefits of similar dietary patterns, in agreement with the antioxidant and anti-inflammatory diet hypothesis [148,149].

Another more recent study used the HEI (2005 and 2010 editions) diet score and a modified version of the Mediterranean diet score [165] to assess the cross-sectional association of these two diet quality scores with COPD severity (according to GOLD stages) and parameters of lung function (FEV1 and FVC) in 121 patients with stable COPD [155]. Both scores reflected high intakes of fruits, vegetables, wholegrains, PUFAs, MUFA, nuts, legumes, and low intakes of refined grains, red/cured meat (and red meat to white meat ratio), saturated fat, empty calories, and sodium. In particular, the Mediterranean diet score, from its original conception [165] to the latest modifications [166], is intended to capture compliance to the plant-based eating patterns of olive tree-growing areas of the Mediterranean basin. According to both HEI and Mediterranean diet scores, the diet quality of the study subjects appeared to need improvements. Although not reaching significance, reduced HEI and Mediterranean diet scores were observed with increased COPD severity, mostly stage 4, and a one-unit increase of the Mediterranean diet score was significantly associated with 2.9 (*p* = 0.002) and 2.8 (*p* = 0.007) increase of FEV1 and FVC, respectively [155]. Although obtained in a small sample of already diseased subjects, these results further suggest protective effects on lung function by the Mediterranean diet pattern. Further high-powered confirmatory studies as well as the evaluation of diet effect on COPD progression over time are highly warranted.

In a very recent cross-sectional study conducted in middle-aged healthy subjects at low-to-moderate CV risk but without pulmonary diseases (Ilerda Vascular Project, ILERVAS) [156], low adherence to the Mediterranean diet as well as low physical activity practice were independently associated with the presence of impaired spirometric values and with ventilatory defects, compared with high adherence to the Mediterranean diet and vigorous physical activity. Therefore, although we are still awaiting interventional studies providing causality, these results agree with those previously obtained with dietary pattern analysis [153] and collectively suggest a beneficial association between the Mediterranean diet and lung function with relevance to both the prevention of respiratory diseases as well as the improvement of COPD.

In a large prospective Asian cohort study, adherence to several recommended dietary patterns as reflected in the AHEI-2010, the alternate Mediterranean diet score, the dietary approaches to stop hypertension (DASH) score, and the healthy diet indicator (HDI), and based on healthy plant-based foods and fish, was associated with a substantially lower risk of 17-year all-cause and disease-specific (CVD, cancer and respiratory disease) mortality, specifically with a 14–28% lower risk of mortality for respiratory diseases [167]. Interestingly, COPD was one of the predominant respiratory conditions contributing to respiratory disease mortality in the study cohort. These results agree with earlier reports of an inverse association between intake of single dietary components of these dietary patterns, such as fruits, and COPD mortality [53]. Other studies in different populations confirmed the beneficial association between adherence to the DASH diet and COPD risk [168], and adherence to the healthy Dutch dietary guideline and the risk of all-cause mortality and COPD development [169].

**Figure 2:**
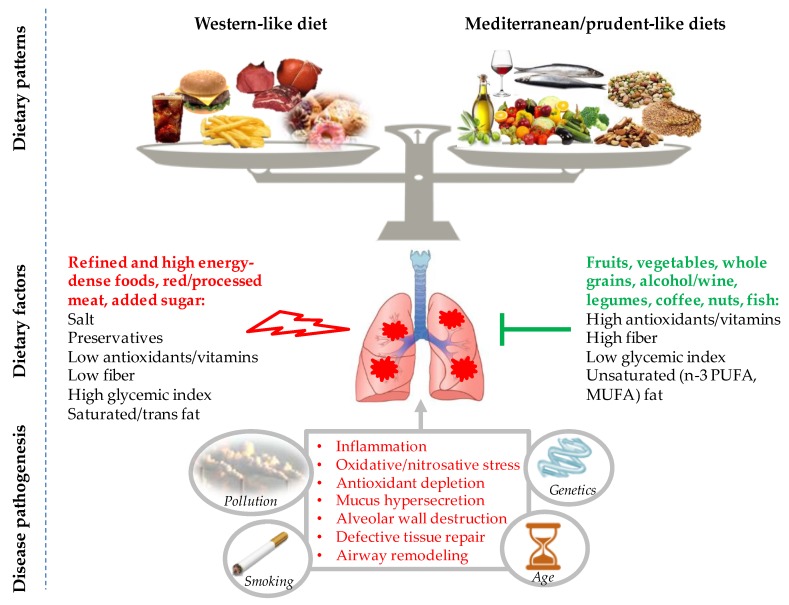
A framework model of the interactions of diets and dietary factors with lung function and COPD development and progression.

Collectively, although needing further confirmations, published studies concordantly suggest a significant role for high-quality whole diet on lung function outcomes and COPD incidence and prevalence, as well as on mortality. Adhering to dietary patterns resembling the general principles of the Mediterranean diet and the prudent diets, which emphasize a variety of healthy plant-based foods (vegetables, fruit, nuts, wholegrains) and fish, avoidance of heavy alcohol intake, and low consumption of foods typical of Westernized patterns (red/processed meat, refined grains, sweets/desserts), exerts beneficial effects in contrast with Western diets (Figure 2). Interestingly, the Western diet has been shown to be positively associated while the prudent/Mediterranean diet inversely associated with serum levels of inflammatory markers [170,171]. Moreover, beneficial effects have also been documented for bioactive nutrients of the healthy diets, such as polyphenols and PUFAs, against visceral adiposity and related inflammation/oxidative stress, mitochondrial dysfunction, as well as insulin resistance [172], thus potentially providing the opportunity to favorably manage the risk associated with metabolic derangements observed in some patients with COPD (obesity and/or abdominal visceral adiposity).

In addition to directly improving inflammation, oxidative stress, and immune and metabolic deregulation, dietary factors may act by inducing modification of gut microbiota, which can influence immune system, systemic inflammation, and metabolism through the production of locally- and systemically-active metabolites, such as the fiber fermentation-derived short chain fatty acids (SCFAs) (butyrate, propionate and acetate) or the carnitine/choline-derived trimethylamine-N-oxide (TMAO). Human studies have found that plant-based diets such as the Mediterranean diet (rich in fiber) shaped the composition of gut microbiota so as to increase the circulating levels of anti-inflammatory SCFAs, while animal-based diets such as the Western diet (rich in carnitine and choline from meat, egg yolks, and high-fat dairy products) were associated with increased levels of the pro-inflammatory TMAO, which is linked to risk of atherosclerosis, CV disorders, and mortality [173]. An emerging concept and potential therapeutic opportunity for dietary modulation is the gut–lung axis, where intestinal microbial modulation can influence the respiratory system [174]. Indeed, high dietary fiber intake was shown to protect against inflammatory airways disease via systemic SCFAs [175]. Moreover, increased circulating TMAO levels were associated with long-term all-cause mortality in patients with COPD [176]. Of course, further studies are required to address whether gut–lung axis modulation by nutrition would beneficially impact lung function and the risk or evolution of COPD.

## 8. Conclusions and Perspectives

Given the alarming increasing burden of COPD worldwide, identification of modifiable risk factors for prevention and treatment of COPD is highly in demand. Based on the available evidence, greater awareness of diet and dietary factors influencing respiratory health may be of interest for public health due to their disease-modifying effects. Many studies in the general population and in subjects with respiratory disease have reported that current dietary habits are qualitatively poor and therefore leave plenty of opportunities for improvements and interventions. Taking into account the increasing smoking habit in developing countries and the worldwide unstoppable phenomenon of Westernization of lifestyle factors, including a more processed and convenience-orientated diet, a two-hit lifestyle burden (smoking and unhealthy diet) is currently rising.

Based on strong evidence of association with improved cardiometabolic health, including lower risk of CV disease, diabetes, and obesity, many scientific organizations recommend the prudent/Mediterranean-like diets as healthy dietary patterns. Published studies also consistently show the adverse effects of the Western diet, rich in refined foods, saturated fat, meat, and sugar, on lung function and the risk of COPD, and, by contrast, the ability of specific dietary factors and diets, mostly the prudent/Mediterranean-like diets loaded by plant-based foods and healthy fats, to preserve lung function and prevent COPD or its evolution over time. Interestingly, the magnitude of effect of diet on lung function is estimated to be comparable to that of chronic smoking [51], underscoring that healthy dietary approaches may have a great impact jointly on COPD development and the associated metabolic and CV risk.

Interestingly, in many studies, specific dietary patterns and/or nutrients exerted benefits on lung function and the risk of COPD, but not asthma, strongly suggesting a true underlying effect rather than a generalized and most probably confounded effect. COPD as well as CV diseases share a systemic inflammatory pathogenesis differently from the immune pathogenesis of asthma. Nutritional targeting of oxidative balance and overwhelming inflammation may therefore represent a unique opportunity to prevent/treat COPD and its related CV co-morbidities.

Of course, there is not a single diet identified as a magic pill for respiratory health. Food groups, including fruits, vegetables, fish, and wholegrain products, contributing to basic nutraceutical ingredients, such as antioxidants, vitamins, fiber, and PUFAs, may vary across the dietary patterns documented to be beneficial for lung function, according to the populations studied. However, some unifying principles of all the healthful diets may be recognized and emphasized in designing preventive nutritional measures. In many studies, dietary factors documented to improve several processes (inflammation, oxidative stress ad immune dysfunction) and co-morbidities (CVD, obesity) of respiratory diseases translate into improved respiratory outcomes. Importantly, considering the early origin of COPD [10] and the profound impact of diet on lung function and later respiratory health, nutrition intervention offers the opportunity for early strategies of primary prevention and/or targeted early therapeutic approaches.

The negative results of supplementation trials with single antioxidant nutrients suggest that the effect of single nutrients may be too small to be observed, reinforcing the notion that combination of nutrients and foods within a dietary pattern may allow cumulative/synergic effects to become apparent. Moreover, since diet tends to track throughout life, this means that exposure to (or lack of) certain nutrients may occur over a long period of time (as captured in observational studies). Therefore, observational studies of dietary benefits may not always translate into positive results from clinical trials (performed in a specific age group with single nutrients and for a limited follow-up).

With regard to disease treatment, several therapeutic strategies, including smoking cessation, pharmacological interventions, and rehabilitation programs, are implemented in COPD patients to improve quality of life, decelerate lung function decline, and prevent major complications. As malnutrition associated with skeletal muscle impairment is an important systemic and disabling consequence of COPD, nutritional support (e.g., energy-enriched diet) has been recently suggested as a valuable adjuvant tool in the management of COPD patients at risk of malnutrition, mainly in combination with physical exercise [177], suggesting that at least some of the adverse functional consequences of severe COPD are reversible by nutritional support [20].

More animal experimentations and human intervention studies are needed to confirm the effectiveness and mechanisms of diet in preventing and treating COPD. A new area of investigation is the microbiota modulation by diet, which offers the prospect of increasing health and mitigating disease risk. However, the field of nutrigenetics (i.e., the relationship between genetic variants and diet) also deserves attention to address the inter-individual variability in response to diet and improve a personalized nutrition intervention to prevent/treat COPD. Moreover, it is possible that diet influences may be different across different clinical phenotypes of COPD, as some evidence suggests [101]. Therefore, more studies are needed to ascertain the effect of diet on different COPD phenotypes and to develop tailored nutritional strategies. Although evidence for the role of diet in COPD is clearly available, smoking cessation and the appropriate pharmacologic therapy remain key measures for the prevention and treatment of COPD.

Although current nutritional guidelines for COPD management do not formally include specific dietary recommendations other than nutritional counseling for malnourished patients, the available scientific evidence provides new directions for future research to substantiate the role of nutrition in lung function maintenance, respiratory disease prevention, and treatment, leading to the ultimate positioning of nutrition on the roadmap to optimal respiratory health.

Based on the evidence presented in this review and pending further evidence from basic science and interventional studies, a pragmatic view for managing respiratory health and COPD, as well as many other aspects of health, would be to recommend (along with physical exercise) a healthy balanced diet characterized by high consumption of fresh fruits, vegetables, wholegrains, plant oils and fish, low intake of alcohol (preferably wine), and avoidance of processed, refined, high-saturated fat foods, sweets, cured/red meats, and sugar-containing beverages.

Most importantly in current times, there may be a wide range of appropriate approaches to diet that should be considered and include social, cultural, and psychological aspects of eating. Moreover, nutrition-based measures for health maintenance and disease management are complicated by issues such as food production and processing technologies that need careful attention and convergent efforts by health policy makers, the food industry, health professionals, and consumers, in order to align nutritional health also with a sustainable food system. All these considerations and achievements have the great potential to improve evidence-based public health recommendations for a healthier eating pattern to adopt early in life as part of a healthy lifestyle in order to preserve lung function and prevent or improve COPD, in addition to encouraging smoking avoidance or cessation, and especially in smokers who are unable to quit smoking.

## Figures and Tables

**Table 1:** Main findings from epidemiological studies linking dietary patterns to adult lung function and chronic obstructive pulmonary disease (COPD) (incidence, prevalence, and severity).

Dietary Patterns	Country (Cohort)	Design (Follow-Up)	Population	Sex (Age)	Diet Assessment Method	Outcome	Outcome Assessment	Main Results	Ref
*Data-driven dietary patterns*									
Meat–dim sum pattern and vegetable–fruit–soy pattern	China (SCHS)	P (5.3 year)	General population *n* = 52,325	F, M (45–74 year)	FFQ and PCA	New onset of cough with phlegm	Self-reported	The meat-dim sum pattern was associated with increased incidence of cough with phlegm (fourth vs. first quartile, OR = 1.43, 95% CI: 1.08, 1.89, *p* for trend = 0.02))	[146]
Prudent pattern and Western pattern	USA (HPFS)	P (12 year)	Health professionals *n* = 42,917	M (40–75 year)	FFQ and PCA	COPD incidence	Self-reported	The prudent pattern was negatively (highest vs. lowest quintile, RR = 0.50, 95% CI: 0.25, 0.98), while the Western pattern was positively (highest vs. lowest quintile, RR = 4.56, 95% CI: 1.95, 10.69) associated with COPD risk	[147]
Prudent pattern and Western pattern	USA (NHS)	P (6 year)	Nurses *n* = 72,043	F (30–55 year)	FFQ and PCA	COPD incidence	Self-reported	The prudent pattern was negatively (highest vs. lowest quintile, RR = 0.75, 95% CI: 0.58, 0.98), while the Western pattern was positively (highest vs. lowest quintile, RR = 1.31, 95% CI: 0.94, 1.82) associated with COPD risk	[148]
Prudent pattern and traditional pattern	United Kingdom (HCS)	C	General population *n* = 1391 (F), *n* = 1551 (M)	F, M (mean 66 year)	FFQ and PCA	Primary outcome: FEV1; Secondary outcomes: FVC, FEV1/FVC, COPD prevalence	Spirometry	The prudent pattern was positively associated with FEV1 in M and F (changes in FEV1 between highest vs. lowest quintiles, 180 mL in M, 95% CI: 0.00, 0.16, p for trend<0.001, and 80 mL in F, 95% CI: 0.26, 0.81, p for trend = 0.008), and negatively with COPD in M (top versus bottom quintile, OR = 0.46, 95% CI: 0.26, 0.81, *p* = 0.012)	[149]
Prudent pattern, high-CHO diet, Western pattern	Swiss (SAPALDIA)	C	General population *n* = 2178	F, M (mean 58.6 year)	FFQ and PCA	FEV1, FEV1/FVC, FEF25-75, COPD prevalence	Spirometry	The prudent pattern was positively associated with lung function and negatively with COPD prevalence (NS)	[150]
Western pattern and prudent pattern	USA (ARIC)	C	General population *n* = 15,256	F, M (mean 54.2 year)	FFQ and PCA	Respiratory symptoms (cough, phlegm, wheeze), FEV1, FEV1/FVC, COPD prevalence	Spirometry	The Western pattern was associated with higher prevalence of COPD (fifth vs. first quintile: OR = 1.62, 95% CI: 1.33, 1.97, *p* < 0.001), respiratory symptoms (wheeze OR = 1.37, 95% CI: 1.11, 1.69, *p* = 0.002; cough OR = 1.32, 95% CI: 1.10, 1.59, *p* = 0.001, phlegm OR = 1.27, 95% CI: 1.05, 1.54, *p* = 0.031), and worse lung function (e.g., percent predicted FEV1: fifth quintile 91.8 vs. first quintile 95.1, *p* < 0.001). The prudent pattern was associated with lower prevalence of COPD (OR = 0.82, 95% CI: 0.70, 0.95, *p* = 0.007), cough (OR = 0.77, 95% CI: 0.67, 0.89, *p* < 0.001), and higher lung function (e.g., percent predicted FEV1: fifth quintile 94.3 vs. first quintile 92.7, *p* < 0.001)	[151]
Cosmopolitan pattern, traditional pattern, and refined food dietary pattern	Netherlands (MORGEN-EPIC)	C	General population *n* = 12,648	F, M (mean 41 year)	FFQ and PCA	FEV1, wheeze, asthma, COPD prevalence	Spirometry and self-reported symptoms	The traditional pattern was associated with lower FEV1 (fifth vs. first quintile, −94.4 mL, 95% CI:−123.4, −65.5, *p* < 0.001) and increased prevalence of COPD (fifth vs. first quintile, OR = 1.60, 95% CI: 1.1, 2.3, p for trend = 0.001); the cosmopolitan pattern was associated with increased prevalence of asthma (fifth vs. first quintile, OR = 1.4; 95% CI: 1.0, 2.0; *p* for trend = 0.047) and wheeze (fifth vs. first quintile, OR = 1.3, 95% CI: 1.0, 1.5; *p* for trend = 0.001)	[152]
		P (5 y)	General population *n* = 2911	F, M (mean 45 year)	FFQ and PCA	FEV1	Spirometry	The refined food pattern was associated with a nonsignificant greater decline in lung function (−48.5 mL, 95% CI: –80.7, −16.3; *p* for trend = 0.11)	[152]
Alcohol-consumption pattern, Westernized pattern, and MED-like pattern	Spain	C	Smokers with no respiratory diseases *n* = 207	F, M (35–70 year)	FFQ and PCA	Impaired lung function	Spirometry	Alcohol-consumption pattern (OR = 4.56, 95% CI: 1.58, 13.18, *p* = 0.005) and Westernized pattern (in F) (OR = 5.62, 95% CI: 1.17, 27.02, *p* = 0.031) were associated with impaired lung function; a nonsignificant trend for preserved lung function was found for MED-like pattern (OR = 0.71, 95% CI: 0.28, 1.79, *p* > 0.05)	[153]
*Diet quality scores*									
Alternate Health Eating Index (AHEI)	USA (NHS and HPFS)	P (16 y NHS; 12 y HPFS)	Nurses *n* = 73,228 (NHS) Health professionals *n* = 47,026 (HPFS)	F (30–55 year), M (40–75)	FFQ and diet quality index (AHEI-2010)	COPD incidence	Self-reported	A higher AHEI-2010 diet score was associated with lower COPD risk (for the fourth fifth of the score, HR = 0.67, 95% CI: 0.53, 0.85, *p* for trend <0.001)	[154]
Health Eating Index (HEI) and MED diet score	Iran	C	Stable COPD *n* = 121	F, M (mean 66.1 year)	FFQ and diet quality index (HEI, and MED score)	COPD severity	Spirometry	Higher MED score was associated with lower FEV1 and FCV. MED score and AHEI decreased as COPD severity increased (NS)	[155]
MED diet score	Spain (ILERVAS)	C	General population *n* = 3020	F (50–70 year), M (45–65 year)	FFQ and MED score	FEV1, FVC, FEV1/FVC	Spirometry	A lower MED diet score was associated with impaired lung function in F (low vs. high adherence, OR = 2.07, 95% CI: 1.06, 4.06, *p* = 0.033) and the presence of obstructive ventilator defects in M (low vs. high adherence, OR = 4.14, 95% CI: 1.42, 12.1, *p* = 0.009)	[156]

Abbreviations: AHEI = Alternate Healthy Eating Index; ARIC = atherosclerosis risk in communities; C = cross-sectional; CHO = carbohydrate; CI = confidence interval; BMI = body mass index; F = female; FEF25-75 = forced expiratory flow at 25-75%; FEV1 = forced expiratory volume in one second; FFQ = food frequency questionnaire; FVC = forced vital capacity; HCS = Hertfordshire cohort study; HEI = Healthy Eating Index; HPFS = Health Professionals Follow-up Study; HR = hazard ratio; ILERVAS = Ilerda vascular project; M = male; MED = Mediterranean; MORGEN-EPIC = Monitoring Project on Risk Factors and Chronic Diseases in the Netherlands—European Prospective Investigation into Cancer and Nutrition; NHS = Nurses’ Health Study; NS = not significant; OR = odds ratio; P = prospective; PCA = principal component analysis; RR = relative risk; SAPALDIA = Swiss Cohort Study on Air Pollution and Lung and Heart Diseases in Adults; SCHS = Singapore Chinese Health Study.
